# Efficacy and safety of Chinese herbal for carotid atherosclerosis: A protocol for systematic review and network meta-analysis

**DOI:** 10.1097/MD.0000000000027909

**Published:** 2021-11-24

**Authors:** Haitao Li, Hongwei Zhi, Xiying Xu, Yahan Wang, Shuai Zhang, Sishuo Zhang

**Affiliations:** a Affiliated Hospital of Shandong University of Traditional Chinese Medicine, Jinan, Shandong Province, China; b First College of Clinical Medicine, Shandong University of Traditional Chinese Medicine, Jinan, Shandong Province, China.

**Keywords:** carotid atherosclerosis, Chinese herbal, network meta-analysis, protocol

## Abstract

**Background:**

Carotid atherosclerosis (CAS) can cause acute events such as myocardial infarction and stroke, seriously injuring human health. There are some shortcomings for statins and surgical in the treatment of CAS. Research has proved that Chinese herbal shows its unique advantages with the multichannel and multitarget treatment strategy. As a result, we propose this study to evaluate the efficacy and safety of Chinese herbal in the treatment of CAS.

**Method:**

We will retrieve the relevant databases to collect the studies of Chinese herbal treatment of CAS up to July 2021. The retrieval language is limited to Chinese and English. Researchers will be responsible for screening studies and extracting data, and use STATA16.0 and WinBUGS1.4.3 for data analysis. We will conduct a bias risk assessment based on the Cochrane Collaboration's bias risk assessment tool and use the grading of recommendations assessment development and evaluation tool to assess the confidence of cumulative evidence.

**Results:**

The study will evaluate the efficacy and safety of Chinese herbal in the treatment of carotid atherosclerosis.

**Conclusion:**

The study will offer more evidence for the treatment of CAS with Chinese herbal and expand the selection range of clinicians.

**Protocol registration number:**

INPLASY2021100112.

## 1 Introduction

Carotid atherosclerosis (CAS) is a common disease that endangers human health and is a significant risk factor for cardiovascular and cerebrovascular diseases.^[1,2]^ Carotid plaques shedding, rupture, and thrombosis can cause acute events such as myocardial infarction and stroke, which brings a huge burden to the patient's family and high financial costs to the society. The survey reveals that the prevalence of CAS is high. In 2020, about 28% of the global population aged 30 to 79 had abnormal carotid intima-media thickness (IMT) of ≥1.0 mm, equivalent to >1 billion people. The proportion of carotid plaques and carotid stenosis was estimated to be 21.1% and 1.5%.^[3]^ Studies have shown that for every 10 μm/y reductions in the thickness of the carotid artery intima-media, the relative risk of cardiovascular disease was reduced by approximately 9%.^[4]^ Therefore, actively preventing and treating CAS is of great significance to prevent cardiovascular and cerebrovascular events.

CAS lesions mainly include carotid intima-media thickening, arterial plaques formation, arterial stenosis, or occlusion. At present, the pathogenesis of CAS is not completely clear, mainly including lipid infiltration, inflammatory response, vascular endothelial injury, and platelet activation. Various studies have shown that low-density lipoprotein (LDL) rich in cholesterol is directly related to the development of atherosclerosis. Ross R first proposed that CAS is a chronic inflammatory disease.^[5]^ Lymphocytes, monocytes, and LDL in the blood can adhere to the injury of endothelial cells in the arterial wall and accumulate to plaques through mechanisms such as changes in endothelial permeability, intercellular transport, and the promotion of inflammatory factors.^[6,7]^ Vascular endothelial dysfunction is an essential link in atherosclerotic vascular disease.^[8]^ In the early stage of atherosclerosis, various risk factors can induce oxidative stress, activate cytokines and increase the level of LDL. At the same time, macrophages migrate to the vascular wall, and inflammatory stimuli promote endothelial dysfunction.^[9]^ After migration, macrophages transform into foam cells, which work with endothelial inflammatory mediators to promote smooth muscle cell proliferation, vascular bed damage, and plaques formation.^[10,11]^ Then, exposed collagen and inflammatory mediators quickly activate platelets and adhere to damaged blood vessels to form thrombosis. ^[12,13]^

The treatment of CAS is mainly statins. Statins belong to hydroxymethylglutaryl-CoA reductase inhibitors, and their lipid-lowering mechanism is the same. However, different types of statin drugs have been derived due to the differences in chemical structure and drug properties. Clinical studies have confirmed that statins can reduce carotid IMT and prevent the formation of new arterial plaques by reducing the LDL content in the blood.^[14–16]^ High-dose statins can significantly reduce the content of macrophages in atherosclerotic lesions, inhibit plaques inflammatory response, stabilize plaques, but cannot eliminate the formed plaques.^[17,18]^ Long-term use of statins can control the growth rate of carotid plaque and delay the occurrence of cardiovascular and microembolization events.^[19,20]^ For patients with carotid stenosis, surgical intervention is necessary to relieve the stenosis. carotid endarterectomy and carotid artery stenting (CAS) are the main surgical methods.^[20–22]^ Surgical treatment can significantly reduce the incidence of stroke,^[23]^ but there are certain risks and complications, such as cranial nerve injury, cardiovascular events, cerebral hyperperfusion syndrome, infection, and postoperative restenosis is also significant problems.^[24]^

Chinese herbal has the characteristics of multichannel and multitarget, and has unique advantages in the clinical prevention and treatment of CAS. Berberine, the main component of HuangLian, can play a protective role in CAS through 4 significant mechanisms: reducing blood lipid, antioxidation, anti-inflammatory, and protecting vascular endothelium.^[25–28]^ DanShen includes salvianolic acid B, salvianolic acid A, and other components. Salvianolic acid B can protect vascular endothelial cells and has an excellent anti-inflammatory effect.^[29,30]^ Salvianolic acid A can significantly improve dyslipidemia and inhibit platelet activation and inflammatory factor expression, thereby preventing the progress of CAS.^[31,32]^Angong Niuhuang Pill can inhibit chronic inflammation, reduce plaque collagen fibers and play an anti-CAS role in the early and middle stages.^[33]^ Liuwei Dihuang Decoction can inhibit platelet aggregation and play a role in anti-CAS.^[34]^ Purified Xuefu Capsule can improve hemodynamics, eliminate atherosclerotic plaque, and has a particular effect on the prevention and treatment of CAS.^[35]^

More and more people recognize the prevention and treatment of CAS by Chinese herbal, but there is no comprehensive comparative study. This study used the means of network meta-analysis (NMA) to evaluate and synthesize the literature related to the treatment of carotid atherosclerosis with Chinese herbal to obtain evidence of its clinical efficacy and safety of carotid atherosclerosis.

## 2 Methods and design

The study will be guided by the Preferred Reporting Items for Systematic Review and Meta-Analysis Protocols (PRISMA-P) guidelines.^[36]^

### 2.1 Study registration

The study has been registered on the International Platform of Registered Systematic Review and Meta-analysis Protocols (INPLASY) with the registration number is INPLASY2021100112 (URL = https://inplasy.com/inplasy-2021–10–0112/).

### 2.2 Inclusion and exclusion criteria

#### 2.2.1 Type of studies

The study will include a randomized controlled trial using Chinese herbal medicine to treat CAS in Chinese or English. Does not include nonrandomized controlled trials, observational studies, animal experiments, and case reports.

#### 2.2.2 Participants

Patients diagnosed as CAS by vascular ultrasound. Age, race, disease severity, and disease duration were not limited.

#### 2.2.3 Interventions

The treatment group received Chinese herbal therapies, including decoctions, Chinese patent medicines, powders, pills, with no restrictions on dosage form, dosage, and usage. The control group received regular western medicine, other conventional treatment, no treatment or placebo. Both 2 groups can be treated with conventional drugs.

##### 2.2.3.1 Types of outcomes

1. Primary outcomes: total carotid plaque area, IMT, crouse plaque score.
2. Secondary outcomes: total cholesterol, triacylglycerol, low-density lipoprotein cholesterol, high-density lipoprotein cholesterol, the incidence of adverse reactions.

### 2.3 Search methods

The study will search PubMed, web of science, Cochrane Central Register of controlled trials, EMBASE, Cochrane Library, China biomedical literature database (SinoMed), China National Knowledge Infrastructure (CNKI), Wanfang database, and VIP database from the beginning to July 2021. In addition, we will search the research registered on the World Health Organization International Clinical Trial Registry Platform (WHO ICTRP) and track the references in the meta-analysis. We will combine medical subject headings (MeSH) and free-text terms to formulate the search strategy. The search strategy for PubMed is summarized in Table 1.

**Table 1:** Search strategy for PubMed.

No.	Search terms
#1	“Carotid atherosclerosis” [MeSH Terms]
#2	Arter∗ Disease∗, Carotid[Title/Abstract] OR Carotid Arter∗ Disease∗[Title/Abstract] OR Artery Disorder∗, Carotid[Title/Abstract] OR Carotid Artery Disorder[Title/Abstract] OR Disorders, Carotid Artery[Title/Abstract] OR Carotid Atherosclero∗[Title/Abstract] OR Atherosclerotic Disease∗, Carotid[Title/Abstract]
#3	#1 OR #2
#4	“Medicine, Chinese Traditional”[MeSH Terms]
#5	Medicine, Chinese Traditional[Title/Abstract] OR Traditional Chinese Medicine[Title/Abstract] OR Traditional Medicine, Chinese[Title/Abstract] OR Zhong Yi Xue[Title/Abstract] OR Chinese Traditional Medicine[Title/Abstract] OR Chinese Medicine, Traditional[Title/Abstract]
#6	#4 OR #5
#7	“Drugs, Chinese Herbal”[MeSH Terms]
#8	Drugs, Chinese Herbal[Title/Abstract] OR Chinese Drugs, Plant[Title/Abstract] OR Chinese Herbal Drugs[Title/Abstract] OR Herbal Drugs, Chinese[Title/Abstract] OR Plant Extracts, Chinese[Title/Abstract] OR Chinese Plant Extracts[Title/Abstract] OR Extracts, Chinese Plant[Title/Abstract]
#9	#6 OR #7 OR #8
#10	“Randomized Controlled Trials as Topic” [MeSH Terms]
#11	“Randomized Controlled Trial” [Publication Type] OR Clinical Trials,Randomized[Title/Abstract] OR Trials,Randomized Clinical[Title/Abstract] OR Controlled Clinical Trials,Randomized[Title/Abstract]
#12	#10 OR #11
#13	#3 AND #9 AND #12

### 2.4 Study collection

Two researchers imported the retrieved literature into Endnote x9 for inspection, screening, and excluded irrelevant literature. If there are differences, they will be resolved through discussion or seeking a third independent researcher.

### 2.5 Data extraction and management

The 2 researchers independently extracted the required data into Microsoft Excel 2019. If the required data are incomplete, we will contact the author for it. If there is no response, the literature will decide whether to exclude it after group discussion. The data we plan to retrieve include several aspects:

#### 2.5.1 Basic information

Title, author, journal, publication date, registration number of the trial registration authority.

#### 2.5.2 Participants

Age, sex, source of subjects, diagnostic criteria, disease severity, sample size.

#### 2.5.3 Intervention information

Treatment method, frequency, dose, and treatment duration.

### 2.6 Outcome indicators

Primary and secondary outcomes, adverse reactions.

#### 2.6.1 Bias risk assessment

The bias risk assessment of 7 aspects of literature will be carried out according to the Cochrane Collaboration's bias risk assessment.^[37]^ The risk is divided into three levels: “low,” “high,” or “unclear.” This assessment is conducted independently by 2 researchers. If there are differences, they will turn to a third researcher for settlement.

#### 2.6.2 Heterogeneity test

Heterogeneity between studies will be assessed using I^2^. When I^2^> 50%, the research is considered to be of apparent heterogeneity, and the difference is discussed. If heterogeneity cannot be ruled out, a random-effects model is used. When *I*^2^ ≤50%, the research is considered homogeneous and needs to be analyzed using a fixed-effects model.

#### 2.6.3 Subgroup analysis

We will analyze the subgroup of patients according to the course of disease, age, and blood lipid level to explore the impact of these factors on the results.

### 2.7 Sensitivity analysis

We will exclude the literature one by one to determine whether the literature has an impact on heterogeneity. If there is no significant change in heterogeneity before and after elimination, the results are stable and reliable. Otherwise, the literature may be a source of heterogeneity, and we will discuss whether to delete the literature.

### 2.8 Statistical analysis

#### 2.8.1 Pairwise meta-analysis

We will use STATA16.0 software for paired meta-analysis. Binary variable data will use odds ratio (OR) as the adequate analysis statistics, and continuous variable data will use weighted mean square deviation (WMD). Besides, each effect quantity is expressed by a 95% confidence interval (CI).

#### 2.8.2 Network meta-analysis

NMA was performed using WinBUGS 1.4.3 and STATA16.0. We use 3 Markov chain Monte Carlo (MCMC) chains for simulation, and set the number of iterations to 50,000, of which the first 20,000 annealing to eliminate the influence of the initial value.^[38]^ We will use trace graph and Brooks-Gelman-Rubin graph to ensure convergence. If the potential scale reduction factor (PSRF) tends to 1, the convergence is better, and the results are more reliable.^[39]^ In addition, we will calculate the surface under the Cumulative Ranking Curve value to rank the interventions, with a value range of 0 to 1. The closer the value is to 1, the better the treatment effect.^[40]^ If there is a closed-loop, a consistency assessment is required. The node splitting method will be used to evaluate the consistency between direct comparison evidence and indirect comparison evidence.^[41]^

### 2.9 Publication bias

Supposing there are >10 researches were included, funnel plots were needed to assess publication bias. There is no apparent deviation if the funnel plots are symmetrical; there may be a deviation if the funnel plots are asymmetric.

### 2.10 Evaluation of evidence quality

The grading of recommendations assessment development and evaluation (GRADE) tool is used to evaluate the quality of evidence from five aspects, which are divided into four levels: high, medium, low and very low.^[42]^

### 2.11 Ethics and communication

The data are from published literature or studies and do not involve patients, so ethical approval is not required.

## 3 Discussions

CAS is the pathological basis of cardiovascular and cerebrovascular diseases and seriously threatens human health. At present, the treatment of CAS is mainly statins. Statins have various effects, such as stabilizing plaque, inhibiting the intensity of local inflammatory reaction and regulating blood lipid. They are widely used in the treatment of CAS, but there are still some harmful curative effects and side effects. A variety of studies have proved that Chinese herbal has the characteristics of multi-channel, multi-target, and more minor adverse reactions. It has a good effect on the treatment of CAS, but there is no comprehensive comparative study. Therefore, the NMA aims to compare the efficacy and safety of different traditional Chinese herbal in treating CAS. There are some unavoidable limitations in this study. For example, first, our research is based on literature data, which may be biased; second, the evaluation criteria of Chinese herbal may be quite heterogeneous. This study hopes to provide more convincing and detailed information for Chinese herbal treatment of CAS and help clinicians make choices.

## Author contributions

**Conceptualization:** Haitao li, Sishuo Zhang.

**Formal analysis:** Shuai Zhang, Sishuo Zhang.

**Funding acquisition:** Shuai Zhang, Sishuo Zhang.

**Methodology:** Hongwei Zhi, Xiying Xu, Shuai Zhang.

**Project administration:** Xiying Xu, Yahan Wang.

**Software:** Hongwei Zhi, Yahan Wang.

**Writing – original draft:** Haitao Li, Xiying Xu.

**Writing – review & editing:** Haitao Li, Sishuo Zhang.
